# Prevalence trends and risk factors associated with HIV, syphilis, and hepatitis C virus among pregnant women in Southwest China, 2009–2018

**DOI:** 10.1186/s12981-022-00450-7

**Published:** 2022-06-27

**Authors:** Shanmei Zhong, Yanyun Ou, Fei Zhang, Zhaosen Lin, Rongye Huang, Aidan Nong, Zhenxian Wu, Huayue Liang, Cai Qin, Qiuyu Wei, Yuan Yang, Dee Yu, Xianyan Tang, Li Ye, Deping Liu, Hao Liang, Bingyu Liang

**Affiliations:** 1 Guangxi Key Laboratory of AIDS Prevention and Treatment, School of Public Health, Guangxi Medical University, Nanning, 530021 Guangxi China; 2 Collaborative Innovation Centre of Regenerative Medicine and Medical BioResource Development and Application Co-constructed by the Province and Ministr, Life Science Institute, Guangxi Medical University, Nanning, 530021 Guangxi China; 3 Chongzuo Center for Disease Control and Prevention, Chongzuo, 532200 Guangxi China; 4 Qinzhou Center for Disease Control and Prevention, Qinzhou, 535000 Guangxi China

**Keywords:** Sexually transmitted infections, Pregnant women, Southwest China, Zero-inflated negative binomial regression

## Abstract

**Objective:**

This study investigated prevalence trends and identified the associated factors of HIV, syphilis and hepatitis C virus (HCV) among pregnant women in the Guangxi Zhuang Autonomous Region (Guangxi), Southwest China.

**Methods:**

Serial cross-sectional surveys were performed annually among pregnant women in Guangxi from 2009 to 2018. Blood specimens were collected to test the prevalence of HIV, syphilis and HCV. Cochran–Armitage analysis was used to assess the trends of HIV, syphilis and HCV prevalence, as well as the sociodemographic and behavioural data. In this study, we used zero-inflated negative binomial (ZINB) regression models to identify factors associated with HIV, syphilis and HCV infection.

**Results:**

A total of 23,879 pregnant women were included in the study. The prevalence of HIV, syphilis and HCV was 0.24%, 0.85% and 0.19%, respectively. There was a decrease in HIV prevalence from 0.54% to 0.10%, a decrease in HCV prevalence from 0.40% to 0.05% and a decrease in syphilis prevalence from 1.53% to 0.30%. The findings based on the ZINB model revealed that pregnant women who had a history of STI had significantly increased risks of HIV (OR 6.63; 95% CI 1.33–32.90) and syphilis (OR 9.06; 95% CI 3.85–21.30) infection, while pregnant women who were unmarried/widowed/divorced were more likely to have HIV (OR 2.81; 95% CI 1.20–6.54) and HCV (OR 58.12; 95% CI, 3.14–1076.99) infection. Furthermore, pregnant women whose husband had a history of STI (OR 5.62; 95% CI 1.24–25.38) or drug use (OR 7.36; 95% CI 1.25–43.43) showed an increased risk of HIV infection.

**Conclusions:**

There was a relatively low prevalence of HIV, syphilis and HCV among pregnant women. Although decreasing trends in HIV, syphilis and HCV infections were observed, effort is needed to promote STI testing in both premarital medical check-ups and antenatal care, especially targeting couples with a history of STI or drug use.

**Supplementary Information:**

The online version contains supplementary material available at 10.1186/s12981-022-00450-7.

## Introduction

Sexually transmitted infections (STI) continue to be a public health concern, especially in developing countries [1–3]. Studies have demonstrated that human immunodeficiency virus (HIV)/acquired immune deficiency syndrome (AIDS), syphilis and hepatitis C virus (HCV) are the most commonly reported STI in pregnant women [4]; their main routes of transmission are sexual exposure and vertical transmission [2]. Physiological changes in pregnant women may increase the susceptibility to STI [5, 6] and result in devastating health consequences [6–8] and adverse pregnancy outcomes [9].

In 2019, about 1.7 million people became newly infected with HIV worldwide, of which 48% were females; also, there were 1.8 million children (0–14 years) living with HIV in 2019 [10]. Most women and children infected with HIV came from developing countries [10]. Globally, there were 6.3 million new cases of syphilis, representing a prevalence of 0.69% among pregnant women, with approximately 355,000 adverse pregnancy outcomes caused by mother-to-child transmission (MTCT) in 2016 [11]. Estimates of the World Health Organisation (WHO) state that approximately 71 million people have chronic HCV infection worldwide [12] and those who are chronically infected will develop cirrhosis or liver cancer. However, the epidemiology of HCV during pregnancy is still poorly documented. The scope of HIV, syphilis, or HCV infection among pregnant women needs to be better understood for resources to be allocated and to improve the quality of life of infants exposed to and/or infected with these viruses.

Although the prevalence of HIV [13, 14], syphilis [15, 16] and HCV [17, 18] among pregnant women has been shown in a few observational studies, there has been no comprehensive and up-to-date study on the epidemiology of these three STI in an area severely affected by HIV/AIDS. To date, studies about these infections are limited and mainly concentrated on the detection [13] or treatment of these infections, pregnancy outcomes [19] and associated factors [20]. Despite several studies reporting that the lack of formal education, inadequate access to healthcare and poverty increase the risk of spreading infectious diseases, mainly in low- and middle-income countries [2, 21], limited information regarding behavioural characteristics of the spread of infectious diseases is available among pregnant women from Southwest China. Identifying the risk factors related to HIV, syphilis and HCV infection among pregnant women could be beneficial for guiding policy formulation and initiating effective targeted preventive interventions in the population. The most cost-effective method to prevent MTCT of STI is routine testing for STI. There are barriers to preventing STI transmission due to a lack of awareness of the risk factors for STI and the extent of the problem. Thus, this study aimed to estimate the prevalence of HIV, syphilis and HCV among pregnant women and identify factors associated with these infections in Guangxi, Southwest China.

## Methods

### Study design and participants

The consecutive cross-sectional study was conducted among pregnant women attending antenatal care between April 2009 and September 2018 in Guangxi, Southwest China. All eligible pregnant women aged from 15 to 49 years-old who self-reported for the first antenatal visit were included after providing informed consent. The following sociodemographic and behavioural data were collected during individual interviews using a standardised questionnaire: age, ethnicity, marital status, education, awareness of HIV-related knowledge, gravidity, parity, multiple sexual partners, history of out-migrating for work and history of STI and drug use (her or her husband) (Additional file 1). HIV/AIDS-related knowledge was assessed using eight basic questions regarding HIV transmission routes, treatment and preventive measures. Those who had a cumulative score of 6 or above were considered as having accurate HIV/AIDS-related knowledge [22, 23].

### STI detection

About 5 ml of peripheral blood was collected from each participant by trained physicians for serological testing. Briefly, blood specimens were tested for antibodies against HIV, syphilis and HCV. HIV antibody testing was performed according to standardized operating procedures based on the manufacturer’s instructions. Any specimen that screened positive by enzyme-linked immunosorbent assay (ELISA) (Wantai Biological Pharmaceutical Co., Beijing, China) was confirmed by western blot (WB) assay (HIV Blot 2.2 WB; Genelabs Diagnostic, Singapore). For syphilis, rapid plasma regain (RPR) (Rongsheng Biotechnical Company, Shanghai, China) was used as a screening test and positive samples were confirmed by Treponema pallidum particle agglutination (TPPA, Serodia; Fujirebio, Fuji, Japan). Subjects with positive results for both RPR and TPPA were considered to have a current syphilis infection. HCV antibody testing was performed using an IgG-based ELISA (Wantai Biological Pharmaceutical Co., Beijing, China).

### Statistical analysis

All data were double entered into EpiData version 3.1 software for Windows (Odense, Denmark) and then transferred to SPSS version 22.0 software (Chicago, IL, USA) and R version 4.2.0 for statistical analysis. The frequencies and percentages for qualitative variables were calculated by year of survey (2009–2018). Cochran–Armitage trend analysis was used to assess the trends of HIV, syphilis and HCV prevalence and risk behaviours.

The zero-inflated negative binomial (ZINB) regression models [24, 25] were used to identify the risk factors associated with three binary outcome variables: HIV, syphilis and HCV infection. As the three STI infections were most commonly 0 and the standard deviation was larger than the mean, the analysis was performed using the pscl package (version 1.5.5) in R. The ZINB regression model can account for the over-dispersion of count data, handle issues related to the presence of many zero values and improve the overall explanatory power by accounting for zero values [26] (Additional file 2: Table S1). Some covariates with few counts were excluded from the ZINB regression model. Crude odds ratios (cOR) from univariate logistic regression models, adjusted odds ratios (aOR) from the ZINB regression model and their respective 95% confidence intervals (95% CI) were presented in a table. The reported *p*-values are two-tailed and deemed statistically significant when *p* < 0.05.

## Results

### Characteristics of participants

A total of 23,879 pregnant women with a mean age of 27.89 ± 5.59 years were recruited to the study. As shown in Table 1, most of the participants were younger than 35 years-old (88.27%). The percentage of married women was much higher (91.81%) than the percentage of single/widowed/divorced women (8.19%). Over half of the participants were non-Han ethnic Chinese (62.32%) and received < 9 years of education (71.48%). Approximately three fifths of pregnant women self-reported being in their second gravidity or more and two fifths of participants self-reported having given birth at least once.

**Table 1:** Demographic characteristics of pregnant women recruited between 2009 and 2018 in southwest of China (n, %)

Variable	Total	2009	2010	2011	2012	2013	2014	2015	2016	2017	2018	cOR (95% CI)	*p* for trend
		(N = 743)	(N = 2773)	(N = 2789)	(N = 2807)	(N = 3200)	(N = 3200)	(N = 3169)	(N = 1195)	(N = 2003)	(N = 2000)		
Age													
< 35 years	21,078	679	2584	2536	2557	2832	2837	2803	1010	1601	1639	1.00	0.00
	88.27%	91.39%	93.18%	90.93%	91.09%	88.50%	88.66%	88.45%	84.52%	79.93%	81.95%		
≥ 35 years	2801	64	189	253	250	368	363	366	185	402	361	1.14 (1.12–1.16)	
	11.73%	8.61%	6.82%	9.07%	8.91%	11.50%	11.34%	11.55%	15.48%	20.07%	18.05%		
Ethnicity													
Other minorities	14,882	72	1678	1691	1680	2052	2196	2090	689	1371	1363	1.00	0.00
	62.32%	9.69%	60.51%	60.63%	59.85%	64.13%	68.63%	65.95%	57.66%	68.45%	68.15%		
Han	8997	671	1095	1098	1127	1148	1004	1079	506	632	637	1.09 (1.08–1.11)	
	37.68%	90.31%	39.49%	39.37%	40.15%	35.88%	31.37%	34.05%	42.34%	31.55%	31.85%		
Marital status													
Married/cohabitating	21,923	711	2628	2555	2592	2959	2942	2926	1046	1795	1769	1.00	0.00
	91.81%	95.69%	94.77%	91.61%	92.34%	92.47%	91.94%	92.33%	87.53%	89.62%	88.45%		
Other	1956	32	145	234	215	241	258	243	149	208	231	0.92 (0.90–0.93)	
	8.19%	4.31%	5.23%	8.39%	7.66%	7.53%	8.06%	7.67%	12.47%	10.38%	11.55%		
Education △													
≥ 9 years	6742	205	690	624	743	760	763	899	497	748	813	1.00	0.00
	28.23%	27.74%	25.06%	22.39%	26.54%	23.87%	23.93%	28.38%	41.84%	37.34%	40.65%		
< 9 years	17,069	534	2063	2163	2057	2424	2426	2269	691	1255	1187	1.10 (1.09–1.11)	
	71.48%	72.26%	74.94%	77.61%	73.46%	76.13%	76.07%	71.62%	58.16%	62.66%	59.35%		

CI means confidence interval, cOR means crude odds ratio, △ means a partial absence of data

### Trends of HIV, syphilis and HCV prevalence

**Fig. 1:**
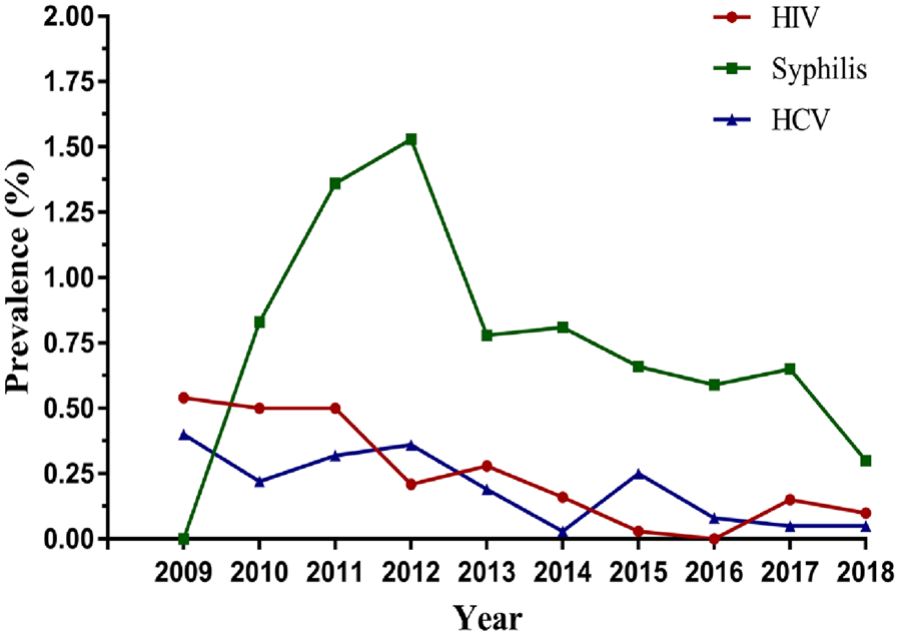
Time trend for prevalence of HIV, syphilis and HCV among pregnant women in southwest of China, 2009–2018

As shown in Table 2 and Fig. 1, the overall HIV, syphilis and HCV prevalence among participants was 0.24%, 0.85% and 0.19%, respectively. There was a significant decrease in the overall HIV, syphilis and HCV prevalence among pregnant women during the study period. The prevalence of HIV reduced from 0.54% to 0.00% during 2009–2018, with a cOR of 0.76 (95% CI 0.67–0.85). The seropositive rate of syphilis increased from 2009 to 2012, with the highest prevalence of 1.53% in 2012, and then fell sharply to 0.3% in 2018 with a cOR of 0.91 (95% CI 0.86–0.97). The prevalence of HCV showed a relatively low level, ranging from 0.40% in 2009 to 0.03% in 2014, with a cOR of 0.83 (95% CI 0.73–0.94).

**Table 2:** Prevalence of HIV, syphilis and HCV among pregnant women in in southwest of China, 2009–2018 (n, %)

Infections	2009	2010	2011	2012	2013	2014	2015	2016	2017	2018	Total	cOR (95% CI)	*p* for trend
	(N = 743)	(N = 2773)	(N = 2789)	(N = 2807)	(N = 3200)	(N = 3200)	(N = 3169)	(N = 1195)	(N = 2003)	(N = 2000)	(N = 23,879)		
HIV+	4	14	14	6	9	5	1	0	3	2	58		
	0.54%	0.50%	0.50%	0.21%	0.28%	0.16%	0.03%	0.00%	0.15%	0.10%	0.24%	0.76 (0.67–0.85)	0.00
Syphilis+	0	23	38	43	25	26	21	7	13	6	202		
	0.00%	0.83%	1.36%	1.53%	0.78%	0.81%	0.66%	0.59%	0.65%	0.30%	0.85%	0.91 (0.86–0.97)	0.00
HCV+	3	6	9	10	6	1	8	1	1	1	46		
	0.40%	0.22%	0.32%	0.36%	0.19%	0.03%	0.25%	0.08%	0.05%	0.05%	0.19%	0.83 (0.73–0.94)	0.00

CI means confidence interval, cOR means crude odds ratio

### HIV/STI-related knowledge and behaviours

This study found that the awareness of HIV-related knowledge was maintained at a high level and increased from 2009 to 2018 (cOR = 1.17, 95% CI 1.15–1.19). During the study period, the percentage of participants who reported a history of STI and drug use decreased (cOR = 0.76, 95% CI 0.71–0.82; cOR = 0.70, 95% CI 0.59–0.84). Concurrently, the percentage of participants who reported their husbands having a history of STI and drug use also decreased (cOR = 0.76, 95% CI 0.72–0.80; cOR = 0.90, 95% CI 0.83–0.98). However, the percentage of participants who reported having multiple sexual partners remained at a low level (0.51%) and did not show a significant decrease (cOR = 0.93, 95% CI 0.87–1.00). Also, the percentages of multi-gravidae and multi-parity gradually increased (cOR = 1.15, 95% CI 1.14–1.16; cOR = 1.17, 95% CI 1.15–1.18) (Table 3).

**Table 3:** HIV/STI-related knowledge and behaviours for pregnant women during 2009 and 2018 in southwest of China (n, %)

Variable	Total	2009	2010	2011	2012	2013	2014	2015	2016	2017	2018	cOR (95% CI)	*P* for trend
		(N = 743)	(N = 2773)	(N = 2789)	(N = 2807)	(N = 3200)	(N = 3200)	(N = 3169)	(N = 1195)	(N = 2003)	(N = 2000)		
Awareness of HIV-related knowledge													
No	2227	204	455	325	258	253	218	125	41	133	215	1.00	0.00
	9.33%	27.46%	16.41%	11.65%	9.19%	7.91%	6.81%	3.94%	3.43%	6.64%	10.75%		
Yes	21,652	539	2318	2464	2549	2947	2982	3044	1154	1870	1785	1.17 (1.15–1.19)	
	90.67%	72.54%	83.59%	88.35%	90.81%	92.09%	93.19%	96.06%	96.57%	93.36%	89.25%		
Primigravidae△													
Yes	9301	373	1268	1413	1257	1362	1171	1168	335	475	479	1.00	0.00
	38.95%	50.20%	45.76%	50.66%	44.78%	42.56%	36.59%	36.86%	28.03%	23.71%	23.95%		
No	14,576	370	1503	1376	1550	1838	2029	2001	860	1528	1521	1.15 (1.14–1.16)	
	61.05%	49.80%	54.24%	49.34%	55.22%	57.44%	63.41%	63.14%	71.97%	76.29%	76.05%		
Parity△													
Nulliparous	12,360	439	1749	1754	1689	1746	1539	1621	475	635	713	1.00	0.00
	51.76%	66.41%	66.33%	66.49%	64.34%	58.85%	54.13%	55.92%	43.62%	35.08%	40.53%		
Parous	9568	222	888	884	936	1221	1304	1278	614	1175	1046	1.17 (1.15–1.18)	
	40.07%	33.59%	33.67%	33.51%	35.66%	41.15%	45.87%	44.08%	56.38%	64.92%	59.47%		
Multiple sexual partners△													
No	23,257	710	2671	2693	2672	3061	3149	3139	1180	1995	1987	1.00	0.06
	97.40%	99.30%	99.26%	99.12%	99.37%	99.67%	99.90%	99.59%	99.16%	99.60%	99.35%		
Yes	123	5	20	24	17	10	3	13	10	8	13	0.93 (0.87–1.00)	
	0.51%	0.70%	0.74%	0.88%	0.63%	0.33%	0.10%	0.41%	0.84%	0.40%	0.65%		
A history of STI△													
No	23,700	736	2728	2753	2778	3176	3177	3167	1192	2001	1992	1.00	0.00
	99.43%	99.46%	98.66%	98.78%	99.36%	99.53%	99.53%	99.94%	99.92%	99.90%	99.60%		
Yes	136	4	37	34	18	15	15	2	1	2	8	0.76 (0.71–0.82)	
	0.57%	0.54%	1.34%	1.22%	0.64%	0.47%	0.47%	6.00%	0.08%	0.10%	0.40%		
Husband’s history of STI△													
No	23,501	732	2715	2705	2718	3130	3147	3160	1191	2003	2000	1.00	0.00
	98.65%	99.19%	98.19%	97.13%	97.42%	98.03%	98.71%	99.72%	100.00%	100.00%	100.00%		
Yes	321	6	50	80	72	63	41	9	0	0	0	0.76 (0.72–0.80)	
	1.35%	0.81%	1.81%	2.87%	2.58%	1.97%	1.29%	0.28%	0.00%	0.00%	0.00%		
A history of drug use△													
No	23,782	735	2741	2780	2785	3190	3196	3166	1187	2002	2000	1.00	0.00
	99.87%	99.73%	99.56%	99.89%	99.75%	100.00%	99.91%	99.91%	100.00%	99.95%	100.00%		
Yes	31	2	12	3	7	0	3	3	0	1	0	0.70 (0.59–0.84)	
	0.13%	0.27%	0.44%	0.11%	0.25%	0.00%	0.09%	0.09%	0.00%	0.05%	0.00%		
Husband’s history of drug use△													
No	23,701	726	2764	2773	2781	3183	3173	3160	1191	1966	1984	1.00	0.01
	99.58%	97.84%	99.75%	99.60%	99.36%	99.59%	99.78%	99.87%	99.83%	99.14%	99.80%		
Yes	99	16	7	11	18	13	7	4	2	17	4	0.90 (0.83–0.98)	
	0.42%	2.16%	0.25%	0.40%	0.64%	0.41%	0.22%	.0.13	0.17%	0.86%	0.20%		

CI means confidence interval, cOR means crude odds ratio. △ means a partial absence of data

### Factors associated with three STIs in zero-inflated negative binomial regression

Tables 4, 5 and 6 show factors associated with a negative binomial count model for HIV, syphilis and HCV infections. HIV infection increased among pregnant women who were unmarried/widowed/divorced (aOR = 2.81, 95% CI 1.20–6.54), who had a history of STI (aOR = 6.63, 95% CI 1.33–32.90) and whose husband had a history of STI (aOR = 5.62, 95% CI 1.24–25.38) or drug use (aOR = 7.36, 95% CI 1.25–43.43). However, being ethnic Han was found to be associated with a reduced risk of HIV infection (aOR = 0.16, 95% CI 0.04–0.72) (Table 4). The probability of having a syphilis infection was higher in the group of pregnant women aged more than 35 years-old compared with those aged below 35 years-old (1.54% vs. 0.75%) (Table 5). ZINB regression analysis identified that pregnant women who aged more than 35 years-old were more likely to be at risk of being infected with syphilis (aOR = 1.64, 95% CI 1.00–2.70). The risk of contracting syphilis was also found to be much higher in pregnant women who were ethnic Han (aOR = 3.44, 95% CI 1.40–8.42) and whose husband had a history of STI (aOR = 9.06, 95% CI 3.85–21.30) (Table 5). Only pregnant women who were unmarried/widowed/divorced (aOR = 58.12, 95% CI 3.14–1076.99) increased the risk of having HCV infection in ZINB regression analysis (Table 6).

**Table 4:** Zero-inflated negative binomial regression model for prevalence of HIV among pregnant women in southwest of China, 2009–2018

Variable	Positive cases (n, %)	Estimate	SE	z value	aOR (95% CI)
Age					
< 35 years	54 (0.26%)				1.00
≥ 35 years	4 (0.14%)	0.17	0.79	0.22	1.19 (0.25–5.58)
Ethnicity					
Other minorities	28 (0.19%)				1.00
Han	30 (0.33%)	− 1.81	0.75	− 2.40	0.16 (0.04–0.72)*
Marital status					
Married/cohabitating	47 (0.21%)				1.00
Unmarried/widowed/divorced	11 (0.56%)	1.03	0.43	2.39	2.81 (1.20–6.54)*
Education level					
≥ 9 years	13 (0.19%)				1.00
< 9 years	45 (0.26%)	0.17	0.40	0.41	1.18 (0.54–2.60)
Awareness of HIV related knowledge					
No	11 (0.49%)				1.00
Yes	47 (0.22%)	− 0.04	0.56	− 0.08	0.96 (0.32–2.86)
Primigravidae					
Yes	22 (0.24%)				1.00
No	36 (0.25%)	0.03	0.48	0.07	1.04 (0.40–2.65)
Multiple sexual partners					
No	48 (0.21%)				1.00
Yes	8 (6.50%)	0.85	0.71	1.20	2.34 (0.59–9.36)
Parity					
Nulliparous	35 (0.28%)				1.00
Parous	18 (0.19%)	0.14	0.61	0.23	1.15 (0.35–3.78)
A history of STI					
No	55 (0.23%)				1.00
Yes	3 (2.20%)	1.89	0.82	2.31	6.63 (1.33–32.90)*
Husband’s history of STI					
No	51 (0.22%)				1.00
Yes	7 (2.18%)	1.73	0.77	2.24	5.62 (1.24–25.38)*
A history of drug use					
No	56 (0.23%)				1.00
Yes	2 (6.45%)	0.89	0.96	0.92	2.43 (0.37–15.99)
Husband’s history of drug use					
No	55 (0.23%)				1.00
Yes	2 (2.02%)	2.00	0.91	2.20	7.36 (1.25–43.43)*

Only OR (95% CI) of count model (negative binomial with “log” link) was shown in the table

*aOR* adjusted odds ratio, *CI* confidence interval

**p < 0.01; *p < 0.05

**Table 5:** Zero-inflated negative binomial regression model for prevalence of syphilis among pregnant women in southwest of China, 2009–2018

Variable	Positive cases (n, %)	Estimate	SE	z value	aOR (95% CI)
Age					
< 35 years	159 (0.75%)				1.00
≥ 35 years	43 (1.54%)	0.49	0.25	1.95	1.64 (1.00–2.70)*
Ethnicity					
Other minorities	116 (0.78%)				1.00
Han	86 (0.96%)	1.24	0.46	2.70	3.44 (1.40–8.42)**
Marital status					
Married/cohabitating	186 (0.85%)				1.00
Unmarried/widowed/divorced	16 (0.82%)	− 0.12	0.36	− 0.32	0.89 (0.44–1.81)
Education level					
≥ 9 years	40 (0.60%)				1.00
< 9 years	162 (0.95%)	0.43	0.25	1.70	1.53 (0.94–2.51)
Awareness of HIV related knowledge					
No	18 (0.81%)				1.00
Yes	184 (0.85%)	− 0.06	0.29	− 0.22	0.94 (0.53–1.67)
Primigravidae					
Yes	47 (0.51%)				1.00
No	155 (1.06%)	0.45	0.25	1.78	1.56 (0.96–2.55)
Multiple sexual partners					
No	92 (0.74%)				1.00
Yes	85 (0.89%)	− 0.04	0.64	− 0.07	0.96 (0.27–3.38)
Parity					
Nulliparous	195 (0.84%)				1.00
Parous	5 (4.07%)	− 0.25	0.24	− 1.03	0.78 (0.49–1.25)
A history of STI					
No	177 (0.75%)				1.00
Yes	25 (18.38%)	2.20	0.44	5.05	9.06 (3.85–21.30)**
Husband's history of STI					
No	199 (0.85%)				1.00
Yes	3 (0.93%)	1.26	0.66	1.91	3.53 (0.97–12.87)
A history of drug use					
No	202 (0.85%)				
Yes	0 (0.0%)^a^	–	–	–	–
Husband’s history of drug use					
No	200 (0.84%)				
Yes	1 (1.01%)^b^	–	–	–	–

Only OR (95% CI) of count model (negative binomial with “log” link) was shown in the table

*aOR* adjusted odds ratio, *CI* confidence interval

**p < 0.01; *p < 0.05

^a, b^The count of this category is equal to or approximately zero and cannot be calculated by the model

**Table 6:** Zero-inflated negative binomial regression model for prevalence of HCV among pregnant women in southwest of China, 2009–2018

Variable	Positive cases (n, %)	Estimate	SE	z value	aOR (95% CI)
Age					
< 35 years	36 (0.17%)				1.00
≥ 35 years	10 (0.36%)	− 0.07	1.01	− 0.07	0.93 (0.13–6.75)
Ethnicity					
Other minorities	25 (0.17%)				1.00
Han	21 (0.23%)	− 0.91	1.34	− 0.68	0.40 (0.03–5.50)
Marital status					
Married/cohabitating	42 (0.20%)				1.00
Unmarried/widowed/divorced	4 (0.20%)	4.06	1.49	2.73	58.12 (3.14–1076.99)**
Education level					
≥ 9 years	10 (0.15%)				1.00
< 9 years	36 (0.21%)	0.61	1.08	0.56	1.84 (0.22–15.37)
Awareness of HIV related knowledge					
No	7 (0.31%)				1.00
Yes	39 (0.18%)	1.15	1.20	0.95	3.14 (0.30–33.35)
Primigravidae					
Yes	10 (0.11%)				1.00
No	36 (0.25%)	− 2.73	1.61	− 1.70	0.07 (0.00–1.52)
Multiple sexual partners					
No	45 (0.20%)				
Yes	0 (0.00%)^a^	–	–	–	–
Parity					
Nulliparous	19 (0.15%)				1.00
Parous	22 (0.23%)	0.08	0.87	0.10	1.09 (0.20–5.95)
A history of STI					
No	46 (0.9%)				
Yes	0 (0.0%)^a^	–	–	–	–
Husband’s history of STI					
No	44 (0.19%)				1.00
Yes	2 (0.62%)	2.58	2.44	1.06	13.24 (0.11–1574.15)
A history of drug use					
No	46 (0.19%)				
Yes	0 (0.0%)^a^	–	–	–	–
Husband s history of drug use					
No	45 (0.19%)				
Yes	1 (1.01%)^b^	–	–	–	–

Only OR (95% CI) of count model (negative binomial with “log” link) was shown in the table

*aOR* adjusted odds ratio, *CI* confidence interval

**p < 0.01; *p < 0.05

^a, b^The count of this category is equal to or approximately zero and cannot be calculated by the model

## Discussion

In this work, we first reported the province-wide prevalence of STI among pregnant women in Southwest China. As far as we know, this study has the largest sample size and longest period, which included 10 years of sentinel surveillance data and provided the trends of three STI among pregnant women from 2009 to 2018 in Guangxi, one of the regions that is strongly affected by HIV infection in Southwest China [27]. The findings of this work are of significance to understanding the STI epidemic and evaluating the strategies of STI prevention and control.

Within this study, the prevalence of HIV, syphilis, HCV and co-infection among pregnant women was less than 1% and there was a decreasing trend of STI, with a small fluctuation in the prevalence of syphilis over time. We found that the decreasing HIV prevalence among pregnant women was consistent with other studies based in China [13, 14, 18]. The HIV prevalence reported in this study was lower than that reported in other countries, such as Cameroon (5.70% in 2017) [28], Ethiopia (4.1% in 2011–2014) [29] and Angola (3.0% in 2017) [30]. Simultaneously, the prevalence of syphilis and HCV was also comparatively lower than reported in other studies [28–32], which was higher than 1% for both.

Several reasons could explain the decreasing trend of STI prevalence among pregnant women in this study. One of the reasons could be that China released the National Implementation Guidelines on the Integrated Prevention of MTCT (iPMTCT) of HIV, syphilis and hepatitis B virus (HBV) programme in 2011. Numbers of comprehensive projects targeting women, combining free premarital medical check-ups and the treatment of STI, were provided in various regions of China during the study period. These effective and profitable programmes might have reduced the burden of STI among pregnant women. As shown in the study results, the increasing awareness of HIV-related knowledge and education, along with the decreasing proportion of pregnant women with a history of STI and drug use, could support this explanation. Another potential reason is the change in the main transmission route of HIV from injected drug use (IDU) to sexual transmission [33]. Moreover, it is possible that the low mobility of this population decreased the potential for spreading infections beyond provincial and national borders.

Our results showed that pregnant women who were unmarried/widowed/divorced were 2.8 times more likely to be infected with HIV than those who were married or cohabiting. A similar study also reported that people engaged in a marital relationship were less exposed to HIV infection [34], probably because individuals are more likely to have multiple sexual partners either before marriage or after divorce or separation [35]. Also, multiple sexual partnerships were independently associated with HIV infection [7]. This study also indicated that pregnant women whose husband had a history of drug use had a positive association with HIV infection. This result can be explained by the fact that some men living in the study site (Guangxi, China) who were infected with HIV via drug use transmitted it to their wife 10 years ago by risky sexual behaviours (e.g., unprotected sex) [36], although the main mode of HIV transmission has shifted from intravenous drug use to sexual transmission [37]. Due to Guangxi being situated along a major heroin trafficking route linking Guangxi with Yunnan and Vietnam, and its close proximity to the world’s major heroin-producing area, known as the Golden Triangle [38], routine HIV screening and treatment for this population are also critical. Policies enhancing HIV management, adherence support and prevention services tailored to their needs, as well as harm reduction services, should be more widely adopted to control the transmission of HIV in this population [39]. With women of reproductive age, identifying the route of HIV infection and transmission is important, but more than half of women did not know all three methods of MTCT (e.g., during pregnancy, during delivery and through breastfeeding) [40]. These results emphasized that it is necessary to promote the awareness of HIV related knowledge, enhance HIV testing in both premarital medical check-ups and antenatal care and promote mother-to-child blocking therapy among HIV-infected pregnant women.

This study showed a higher syphilis prevalence among pregnant women aged ≥ 35 years compared to those aged < 35 years. Women in the higher age groups had more sexual experience and a progressively increasing exposure duration to sexual activity could result in the higher risk of syphilis infection [1, 35]. Previous research has indicated that syphilis could also facilitate HIV transmission by compromising the skin barrier, activating immune cells and increasing viral load [41, 42]. Untreated maternal syphilis during pregnancy can result in a variety of adverse outcomes in both pregnancies and infants [2, 9]; therefore, it is crucial to improve the ability to carry out timely interventions to reduce the spread of syphilis infection. There are several recommendations that we can make. Firstly, early identification, integrated antenatal screening and disease surveillance, as well as early medical treatment of syphilis infection among pregnant women, should be widely implemented. Secondly, syphilis screening is required at the first prenatal care examination and should be repeated at delivery. Thirdly, the local health department should conduct patient-level case management for pregnant women infected with syphilis. Case management involves ensuring necessary treatment and follow-up testing during pregnancy.

In this study, pregnant women and husbands who had a history of STI contributed to HIV infection and pregnant women who had a history of STI contributed to syphilis infection. Nevertheless, the early stages of these infections among pregnant women and their husbands may present with minimal or no symptoms, so they may remain undiagnosed and untreated. Untreated STI among pregnant women could represent a serious risk for survival and may increase the risk of HIV, syphilis and HCV transmission to their infants. Untreated male partners are a critical source of maternal reinfection [43], so it is crucial to provide additional age- and risk-based screening for STI during pregnancy, including regular STI testing in pregnant women as well as their sexual partners.

The present study has several limitations. Firstly, this study relied on self-reported data. As such, the underreporting of behaviours such as multiple sexual partners and drug use due to social stigma, desirability and discrimination may have occurred. Secondly, the low number of positive cases may have limited the ability to identify other relevant risk factors in the study. Thirdly, our study was based on consecutive cross-sectional surveys, which limited the ability to examine causal relationships. Hence, further research is needed to address these limitations.

This is the first study to determine the prevalence of HIV, HCV and syphilis and the risk factors among pregnant women with a large sample size in the cross-border region of Guangxi Province, China. Despite the above-mentioned limitations, the prevalence estimated in this long-term consecutive study was derived from data of sentinel surveillance and might be close to the true prevalence of these three STI among pregnant women in this border area. Nevertheless, further studies on behavioural risk factors that influence the emergence and spread of STI in pregnant women need to be carried out in Guangxi.

## Conclusions

This study has presented the relatively low HIV, syphilis and HCV prevalence with decreasing trends among pregnant women over a 10-year study in Southwest China. Decreasing HIV/STI-related behaviours such as drug use and having a history of STI infection have been reported. Factors such as being older than 35 years, unmarried/widowed/divorced, having a history of STI and having a history of drug use were the main risk factors for STI infection. The results highlighted the fact that integrated interventions involving screening STI before marriage, diagnostic testing and timely treatments should be implemented and help to further reduce new infections in this group, also avoiding vertical transmission.

## Supplementary Information

**Additional file 1.** Maternal quantitative questionnaires.

**Additional file 2: Table S1.** Model fitness for three STI among pregnant women in southwest China, 2009–2018.

## Data Availability

The datasets used or analysed in the study are available from the corresponding author on reasonable request.

## Abbreviations

STI: Sexually transmitted infections
HIV: Human immunodeficiency virus
AIDS: Acquired immune deficiency syndrome
HCV: Hepatitis C virus
MTCT: Mother-to-child transmission
WHO: World Health Organisation
ELISA: Enzyme-linked immunosorbent assay
WB: Western blot
RPR: Rapid plasma regain
TPPA: Treponema pallidum particle agglutination
cOR: Crude odds ratios
aOR: Adjusted odds ratios
CI: Confidence intervals
CDC: Centre for Disease Control and Prevention
ZINB: Zero-inflated negative binomial
