# Supplementary material for: Prevalence trends and risk factors associated with HIV, syphilis, and hepatitis C virus among pregnant women in Southwest China, 2009–2018
Source: AIDS Res Ther. 2022 Jun 27;19:31. doi: 10.1186/s12981-022-00450-7 (PMC9238009; doi:10.1186/s12981-022-00450-7)
Supplement: Supplementary file 1 — Additional file 1. Maternal quantitative questionnaires. [file 12981_2022_450_MOESM1_ESM.docx]

**Maternal quantitative questionnaires**

Introduction:

Hello, We are conducting a survey mainly to investigate your awareness of health knowledge, attitude and behavior. It may involve some of your privacy, but the content of your answers will be kept strictly confidential. We only use these questionnaires to do population analyses, not to do personal analyses. We won't disclose your information to any other individuals or organizations. So we hope you answer the following questions truthfully. This investigation will take you some time. We have to say sorry for any convenience caused. Thank you for your support.

**Questionnaire based information**

| No. | **Questions and options** |
| --- | --- |
| A01 | **Survey location:** ___________ province (autonomous region / municipality) ___________city/district/count**y** |
| A02 | **Informed consent: _______________ (Yes/No)** |
| A03 | **Questionnaire number** □□□□□□□□□□  (administrative division GB code + investigation object category + investigation object number) |
| A04 | **Investigation date** Date: **___________** |

**Socio-demographic, behavioural and psychological questions**

| **No.** | **Questions and options** | **Answers** |
| --- | --- | --- |
| B01 | **Your birthday?** ____ year____month day |  |
| B02 | **Your marital status?** □ unmarried □ married □ cohabit □ divorced or widowed |  |
| B03 | **You registered residence?**  □ local province  □ outside Guangxi province (please note: _____________)  □ other country（please note:______________ ）(Skip to B05) |  |
| B04 | **Your nationality?** |  |
| B05 | **What is your degree of education?**  □ Illiteracy or semi-literate □ Primary school □ Middle school □ High school/ technical secondary school/ vocational high school □ Junior college and above □ Graduate degree and above |  |
| B06 | **What is the gestational age of this pregnancy?** | week(s) |
| B07 | **How many times have you been pregnant so far?** | time(s) |
| B08 | **How many times have you given birth so far ?** | time(s) |
| B09 | **Have you ever been abroad to work or do business?** □ Yes □ No |  |
| B10 | **Has your husband** **ever** **been abroad to work or do business?** □ Yes □ No |  |
| **HIV/AIDS knowledge** | | |
| C01 | **Can you tell if someone is infected HIV from her/his appearance?** □ Yes □ No □ I don't know |  |
| C02 | **Can mosquito bites transmit HIV?** □ Yes □ No □ I don't know |  |
| C03 | **Can we get** **HIV-infected by eating with someone living with HIV?** □ Yes □ No □ I don't know |  |
| C04 | **Does it increase the risk of HIV infection if someone has** **gotten an HIV-infected blood transfusion?**   - Yes □ No □ I don't know |  |
| C05 | **Is it possible to get** **HIV-infected by** **share needles with someone living with HIV?**  □ Yes □ No □ I don't know |  |
| C06 | **Are children born to HIV-infected women at risk of HIV infection?** □ Yes □ No □ I don't know |  |
| C07 | **Can condom use correctly reduce the risk of infecting and transmitting HIV?** □ Yes □ No □ I don't know |  |
| C08 | **Does having sex only with regular partners reduce HIV transmission?** □ Yes □ No □ I don't know |  |
| **Sexual behaviour characteristics** | | |
| D01 | **Do you have sexual partners other than your husband or boyfriend?** □ Yes □ No |  |
| **HIV-risk behaviour** | | |
| E01 | **Does your husband use any illicit drug? Or has your husband ever used illicit drug? (Including heroin, cocaine, opium, marijuana, morphine, methamphetamine, doloretine, K powder (ketamine), ecstasy, and cannabis )** □ Yes □ No |  |
| E02 | **Do you use any illicit drug? Or have you ever used illicit drug? (Including heroin, cocaine, opium, marijuana, morphine, methamphetamine, doloretine, K powder (ketamine), ecstasy, and cannabis )** □ Yes □ No (Skip to F01) |  |
| E03 | **Do you inject illicit drug? Or have you ever injected illicit drug?** □ Yes □ No (Skip to F01) |  |
| E04 | **Did you share needles with others when you inject illicit drug?** □ Yes □ No |  |
| **HIV testing ( self-reported)** | | |
| F01 | **Has your husband been diagnosed with sexually transmitted diseases (STIs)?**  □ Yes □ No □ I don't know |  |
| F02 | **Have you been diagnosed with a sexually transmitted disease?** □Yes □No (Skip to G01) |  |
| F03 | **What kinds of STIs have you been diagnosed with in the past year? (Multiple options)**  □ Gonorrhea □ Syphilis □ Genital Chlamydia trachomatis infection □ Condyloma acuminatum  □ Genital herpes □ Others ( please note: ) |  |
| **This the end of the investigation. Thank you for your cooperation. To understand your sexual health, we would like to collect your 5ml blood for HIV, syphilis and hepatitis C test. The test is free for you, and we will tell you the result of the test.** | | |
| G01 | **Would you like to donate blood and be tested for sexual transmitted disease after the investigation?** □ Yes □ No |  |
| G02a | **Have you ever tested positive for HIV before?** □ Yes □ No (Skip to G03) |  |
| G02b | **If yes, what was the earliest date of the positive test ?** | Date: ______________ |
| **The following questions should be filled out by the investigator based on the test results.** | | |
| G03 | **HIV antibody test results** |  |
|  | **First ELISA screening** □ Positive □ Negative(Skip to G04) |  |
|  | **Second ELISA recheck** □ Positive □ Negative |  |
|  | **Confirmed test** □ Positive □ Negative □ Suspicious □ No detected |  |
| G04 | **Syphilis test result** |  |
|  | **ELISA screening** □ Positive □ Negative (Skip to G05) |  |
|  | **RPR/TRUST detection** □ Positive □ Negative |  |
| G05 | **HCV test result** |  |
|  | **First ELISA screening** □ Positive □ Negative |  |
|  | **Second ELISA re-test** □ Positive □ Negative |  |

**The questionnaire** **quality control table**

| Item | Completion situation | | Note |
| --- | --- | --- | --- |
|  | Yes | No |  |
| Specification, clear |  |  |  |
| All questions were answered, no leakage |  |  |  |
| No logical errors |  |  |  |

Note: Please put “√” in accord with the options

Investigator sign: ____________ Investigation date: __________________

Quality control supervisor sign: ____________ Date: _________________
