# Supplementary material for: Prevalence trends and risk factors associated with HIV, syphilis, and hepatitis C virus among pregnant women in Southwest China, 2009–2018
Source: AIDS Res Ther. 2022 Jun 27;19:31. doi: 10.1186/s12981-022-00450-7 (PMC9238009; doi:10.1186/s12981-022-00450-7)
Supplement: Supplementary file 2 — Additional file 2: Table S1. Model fitness for three STI among pregnant women in southwest China, 2009–2018. [file 12981_2022_450_MOESM2_ESM.docx]

| **Table S1.** Model fitness for three STI among pregnant women in southwest China, 2009–2018 | | | | | | | | |  |
| --- | --- | --- | --- | --- | --- | --- | --- | --- | --- |
| **Model fitness** | **HIV** | |  | **Syphilis** | |  | **HCV** | | |
|  | **logistics regression** | **ZINB regression** |  | **logistics regression** | **ZINB regression** |  | **logistics regression** | **ZINB regression** | |
| -2log likelihood | 703.85 | 615.49 |  | 2153.70 | 1894.65 |  | 658.86 | 568.19 | |
| likelihood ratio test | - | 88.36** |  | - | 259.05** |  | - | 90.67** | |
| AIC | 721.85 | 669.49 |  | 2165.70 | 1940.65 |  | 664.86 | 606.19 | |
| Note: ZINB: Zero-inflated negative binomial regression; AIC: Akaike information criterion; **P<0.01 | | | | | | | | |  |
